# Laparoscopic Conversion of Gastric Plication to One Anastomosis Gastric Bypass

**DOI:** 10.1007/s11695-024-07303-4

**Published:** 2024-06-07

**Authors:** Hadar Nevo, Mohamad Hamoud, Wail Khuri, Shams-Eldin Mokari, Samih Zoabi, Nasser Sakran

**Affiliations:** 1 Department of General Surgery, Holy Family Hospital, P.O. Box 8, Nazareth, Israel; 2 The Azrieli Faculty of Medicine Safed, Bar-Ilan University, Ramat Gan, Israel

**Keywords:** Obesity, Revisional surgery, Gastric plication, One anastomosis gastric bypass

## Abstract

Laparoscopic gastric plication (LPG) is a restrictive procedure to reduce the size of the stomach by folding and suturing the stomach to decrease its capacity. LGP has a high revision rate, mostly to sleeve gastrectomy. To the best of our knowledge, this is the first such report on the bariatric population that has been never described before.

The purpose of this video was to show the feasibility and safety, as well as the main technical aspects, of a laparoscopic conversion of gastric plication to One Anastomosis Gastric Bypass.

**Supplementary Information:**

The online version contains supplementary material available at 10.1007/s11695-024-07303-4.

## Background

Laparoscopic Gastric Plication (LGP) is a surgical procedure designed to reduce the size of the stomach by folding and suturing the stomach to decrease its capacity. This is a restrictive procedure that does not involve removing any part of the stomach or rerouting the digestive tract [1].

Although the complication rate is very low in different studies [2, 3], the mid and long-term results are scant and associated with poor weight loss outcomes [4, 5].

Conversion bariatric surgeries are technically demanding and carry a higher risk of complications when compared to primary procedures [6].

The video aims to demonstrate the main technical aspects, feasibility, and safety of conversion of LGP to one anastomosis gastric bypass (OAGB).

## Methods

**Fig. 1:**
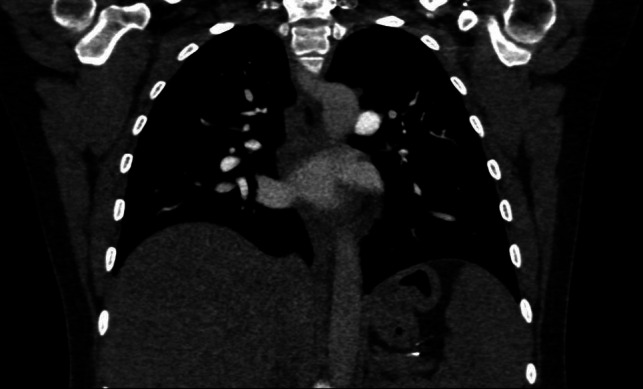
Chest CT scan, showing gastric prolapse

A 31-year-old female underwent Gastric Plication for obesity class II in 2021. Pre-surgery her body mass index (BMI) was 35 kg/m^2^. Two years post-surgery, she was referred to our clinic due to weight recurrence up to BMI 37 kg/m^2^. Her medical history revealed vitamin D deficiency. Upper GI endoscopy revealed normal esophageal, gastric mucosa, and duodenal findings. The stomach body exhibited folds due to plication. One year before the surgery, she also underwent a chest CT scan, showing gastric prolapse (Fig. 1). Due to that, we planned to resect the bypassed stomach from the angulus level.

Surgical treatment options were discussed with the patient including sleeve gastrectomy, Roux-en-Y Gastric Bypass, and OAGB procedure, and a conversion of the LGP to OAGB was scheduled [7].

## Results

**Fig. 2:**
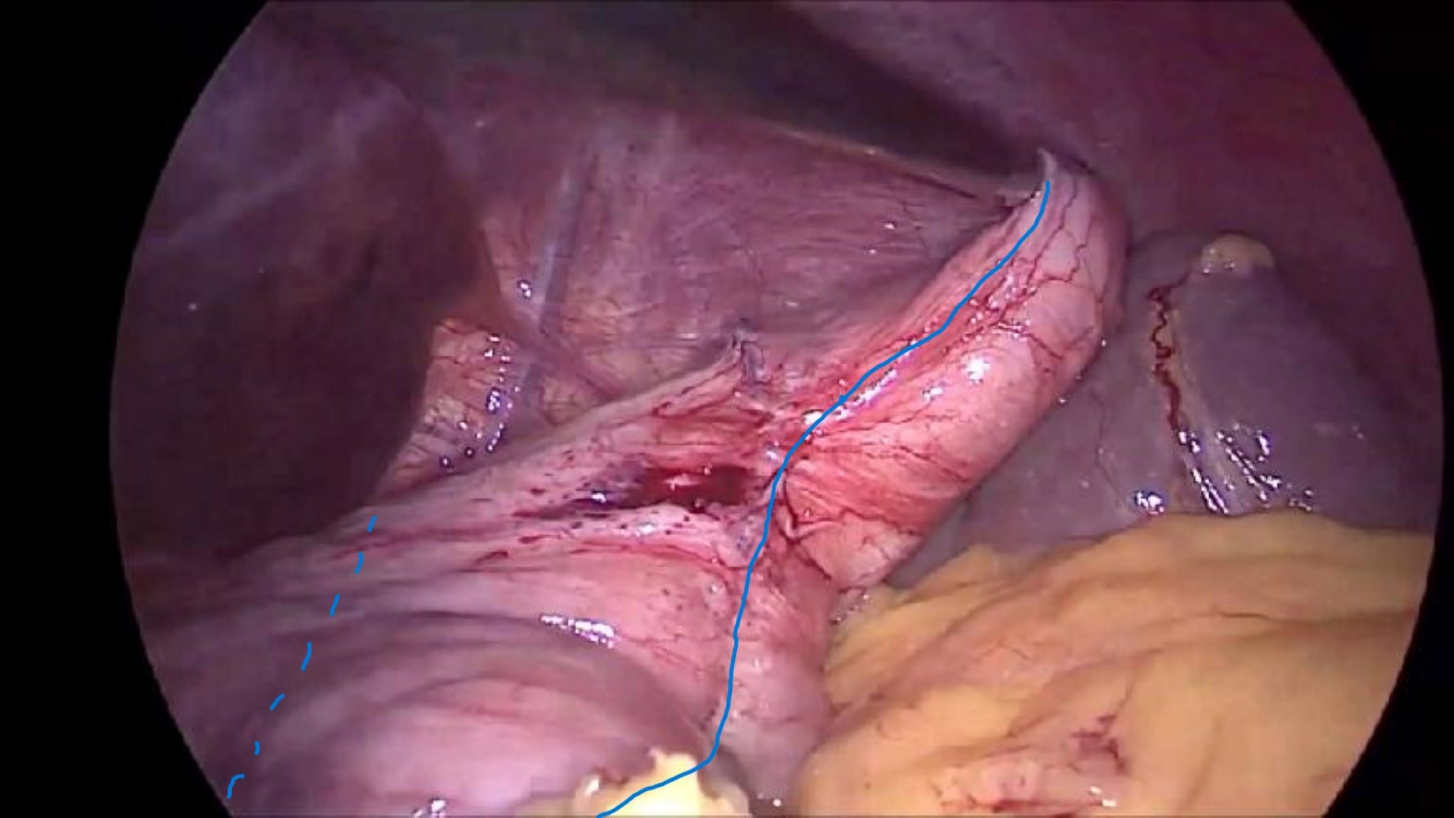
Laparoscopic gastric plication. Plication suture is marked by continuous blue line, and the dashed line is the cute edge of the pouch

**Fig. 3:**
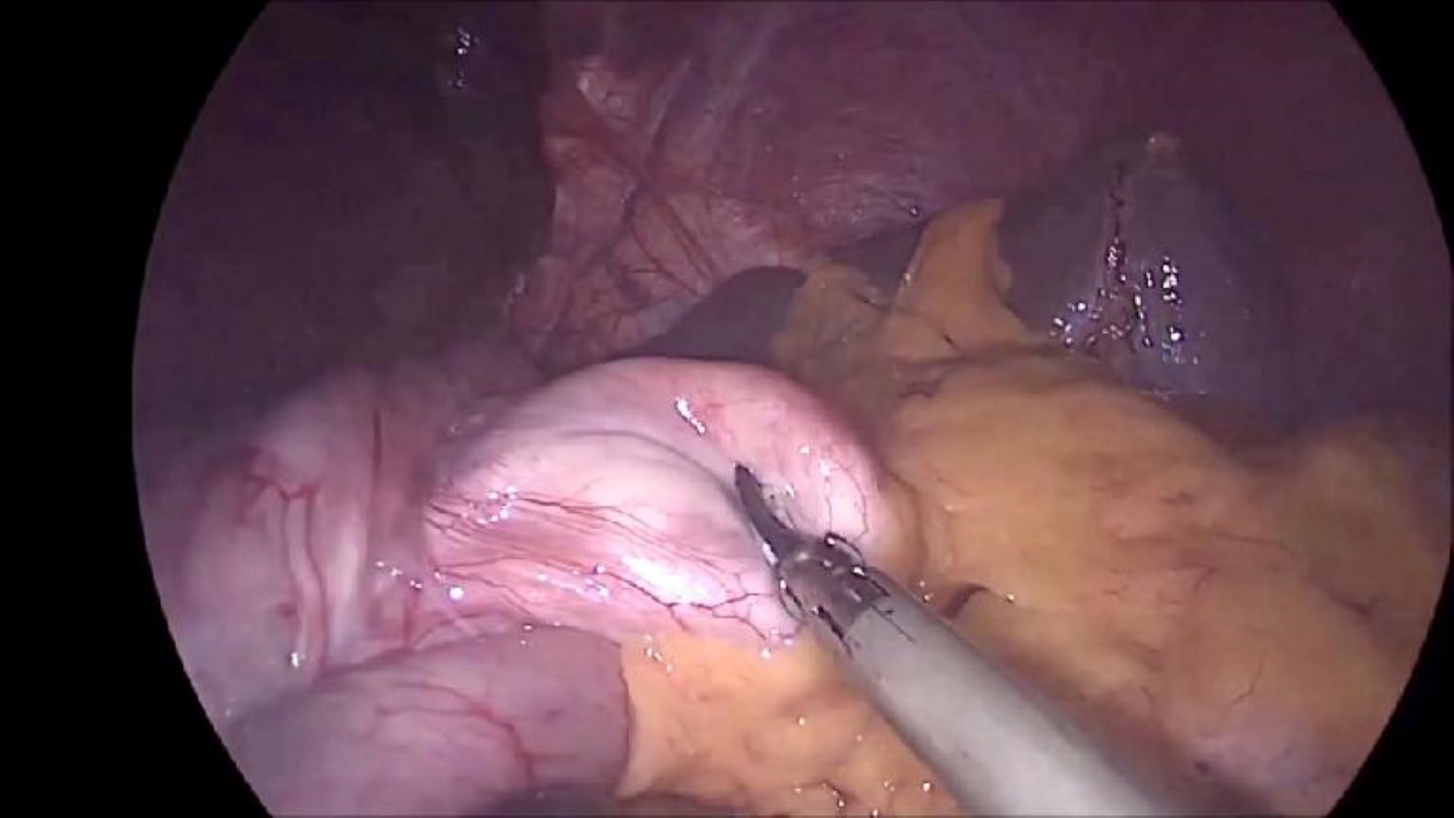
Gastric prolapse, with mobilized fundus

The technical aspects of the procedure are comprehensively described by Sakran et al. study [8]. On inspection, the surgeon observed that the previous imbrication was conducted using non-absorbable sutures. Ensuring that the stomach is not folded during the gastric pouch creation is crucial for the safety of the procedure and may include removing all the plication sutures. In our specific case, the LGB was notably wide (Fig. 2; plication suture is marked by continuous blue line, and the dashed line is the cute edge of the pouch), accompanied by a gastric prolapse (Fig. 3). This circumstance led us to opt for removing only the sutures at the angulus level to ensure an adequate gastric contour. The stapler was positioned medially to the plication line, and the procedure was conducted under direct visualization

A small window was made between the lesser omentum and the lesser curvature of the stomach, entering the lesser sac 2 cm below the angulus.

Using a 45-mm linear stapler, a stapler line was fired horizontally under the crow’s foot. The fundus was adequately mobilized as shown in Figs. 2 and 3. Then, four vertical staplers were fired towards the angle of His, medially to the previous stitches of the plication (Fig. 3), and around a 34-Fr gastric lavage tube, creating a long and narrow pouch. Upon firing the first staler, sufficient stomach should be left to ensure adequate passage of gastric secretions from the proximal to the distal gastric remnant, thus reducing the risk of obstruction at this level. Gastrotomy was then made in the lower right corner of the pouch.

After identification of the ligament of Treitz, a loop of jejunum was antecolically pulled up towards the gastric pouch. Approximately 180 cm distal to the ligament, an enterotomy was created, followed by the performance of an antecolic antegastric loop gastrojejunostomy. The anastomosis was created with a length of 4 cm.

To ensure the safeness of the anastomosis, a 34-Fr gastric lavage tube was passed through the anastomosis into the efferent limb, followed by a methylene-blue test.

The stapler holes were closed by a one-layer running absorbable seromuscular-seromuscular 3.0 V-Loc™ suture to invaginate the proximal staple line.

Finally, fixation of the efferent loop with the bypassed stomach was made with 3.0 V-Loc™ suture. Keeping the afferent loop and gastric pouch higher than the efferent loop in order to create an isoperistaltic conduit.

The operating time was 67 min, exceeding the average duration of OAGB procedure (40–50 min) [8]. This duration reflects the challenges associated with conversion surgeries.

The postoperative course was uneventful, and on 2-year follow-up, the patient had no complaints, and her blood exam showed iron deficiency anemia (11.6 mg/dl). EWL% was 117 with BMI −23 kg/m^2^.

## Conclusion

Conversion of LGP to OAGB is feasible and should be done in a high-volume center and experienced surgeons. Additional data is required to assess the safety of the procedure.

### Supplementary Information

ESM 1 (MP4 408421 kb)

## References

[1] Kourkoulos M, Giorgakis E, Kokkinos C (2012). Laparoscopic gastric plication for the treatment of morbid obesity: a review. Minim Invasive Surg..

[2] Ji Y, Wang Y, Zhu J (2014). A systematic review of gastric plication for the treatment of obesity. Surg Obes Relat Dis..

[3] Albanese A, Prevedello L, Verdi D (2015). Laparoscopic gastric plication: an emerging bariatric procedure with high surgical revision rate. Bariatr Surg Pract Patient Care.

[4] Abdelgawad M, Elgeidie A, Sorogy ME (2022). Long-term outcomes of laparoscopic gastric plication for treatment of morbid obesity: a single-center experience. Obes Surg..

[5] Shen D, Ye H, Wang Y (2013). Comparison of short-term outcomes between laparoscopic greater curvature placation and laparoscopic sleeve gastrectomy. Surg Endosc.

[6] Brethauer SA, Kothari S, Sudan R (2014). Systematic review on reoperative bariatric surgery: american society for metabolic and bariatric surgery revision task force. Surg Obes Relat Dis..

[7] Ramos AC, Chevallier JM, Mahawar K (2020). IFSO (International Federation for Surgery of Obesity and Metabolic Disorders) Consensus Conference Statement on One-Anastomosis Gastric Bypass (OAGB-MGB): results of a modified Delphi study. Obes Surg..

[8] Sakran N, Haj B, Pouwels S (2023). Standardization of the one-anastomosis gastric bypass procedure for morbid obesity: technical aspects and early outcomes. Surg Laparosc Endosc Percutan Tech..

